# A Method to Form Smooth Films of Liquid Metal Supported by Elastomeric Substrate

**DOI:** 10.1002/advs.201800256

**Published:** 2018-08-09

**Authors:** Arthur Hirsch, Stéphanie P. Lacour

**Affiliations:** ^1^ Bertarelli Foundation Chair in Neuroprosthetic Technology Laboratory for Soft Bioelectronic Interfaces Institute of Microengineering Institute of Bioengineering Centre for Neuroprosthetics École Polytechnique Fédérale de Lausanne (EPFL) 1202 Geneva Switzerland

**Keywords:** elasticity, elastomers, electrical conductivity, liquid metals, micropatterning, thin metal films

## Abstract

Several methods are proposed to manipulate and pattern liquid metal films into elastic conductors but all lack precise control over the film thickness and roughness, thereby limiting its uniformity, stability, and reproducibility. Here, an approach relying solely on wetting phenomena is proposed to produce smooth film of liquid gallium (Ga) on extended surface areas with controlled thickness and electrical properties. The surface chemistry and topography of silicone rubber (poly(dimethylsiloxane)) is engineered with microstructured pillars and gold precoating layer to produce Ga superlyophilic substrates. Physical vapor deposition of Ga on such substrates leads to the formation of smooth and homogeneous films by imbibition of the surface topography rather than coalescence and formation of Ga drops. By capillarity, Ga accumulates in between the pillars up to their top surface, forming a smooth film with a root mean square roughness (Rq) smaller than 100 nm. The wetting conditions and electromechanical properties of the resulting films are compared based on the selection of the microtexture patterns and a model of the film sheet resistance as a function of the texture geometrical parameters is established.

The wetting behavior of a liquid to a solid substrate depends both on the chemical affinity of the materials and the surface topology of the substrate. Water is repelled by hydrophobic substrates, beading up to form drops with contact angles larger than 90°. By contrast, it spreads out on hydrophilic surfaces to form extended drops with a contact angle smaller than 90°.^1^ The surface roughness of a solid further influences the wettability of a liquid. Engineering microstructures on a hydrophobic substrate increases the contact angle^2^ and can produce superhydrophobic surfaces for applications such as self‐cleaning surfaces. Similarly, increasing the roughness of a hydrophilic substrate can reduce the effective contact angle and can even lead to the formation of a continuous liquid film through imbibition of the surface roughness.^3^, ^4^

In this Communication, we focus on liquid metal films therefore refer to lyophobic and lyophilic surfaces. Gallium (Ga) is a unique liquid metal. With a melting point of 30 °C and the ability to supercool, Ga can be handled in the liquid state at room temperature. Unlike mercury, it has a near zero vapor pressure^5^ and low toxicity,^6^, ^7^ making it safe to manipulation. Ga and some Ga‐based alloys have recently gathered significant interest from the scientific community as they offer a unique set of electromechanical properties for the design of soft, stretchable, and reconfigurable electronics and robotics,^8^ namely bulk metal electrical conductivity and the ability to reconfigure.

The practical implementation of liquid Ga film conductors for stretchable electronics is challenged by Ga unconventional rheology, which translates in the need for adapted patterning and deposition processes.^9^ In oxygen free environment, Ga does not wet materials like polymers or glass.^10^ The high surface tension of Ga (708 mJ m^−2^)^11^, ^12^ prevents the design of freestanding structures below the capillary length (≈3.5 mm). In the presence of oxygen, however, a solid, passivation oxide skin (≈3 nm thick Ga_2_O_3_ film) spontaneously forms at the surface of Ga. The oxide layer can counterbalance Ga surface tension and enable the creation of nonspherical metastable shapes at length scales below the capillary length.^13^, ^14^ Accordingly, patterning techniques such as 3D printing, spraying or doctor blading rely on the presence of the oxide skin to stabilize the liquid metal in a metastable state after forcing it into a prescribed shape. To date, these approaches do not enable fine control on the Ga film thickness and roughness over large surface area thereby limiting reproducibility over batches of Ga conductors.

We have recently proposed a process relying solely on wetting phenomenon to deposit and pattern continuous thin films of liquid metal on various substrates.^15^, ^16^ The receiving surface is rendered Ga lyophilic via a thin alloying coating preliminarily deposited on the substrate. For example, a thin layer of gold (Au, 60 nm thick) may be sputtered on the substrate prior to thermal evaporation of Ga in vacuum. During this physical vapor deposition process, Ga first condenses and alloys with the Au layer to form AuGa_2_ then accumulates into drops that grow in size and number with the deposition. The resulting film consists of randomly distributed micrometric drops of liquid Ga above an overall thin solid–liquid film. (**Figure** **1** left panel). The film displays good electromechanical behavior with a low sheet resistance (0.5 Ω sq^−1^) and large stretchability (400% uniaxial strain). Adding more Ga, i.e., extending the evaporation process, leads to a rougher surface with enlarged Ga drops. The resulting sheet resistance of the biphasic film therefore does not significantly decrease below 0.5 Ω sq^−1^ as most of the conductive material accumulates in the drops.^15^

**Figure 1:**
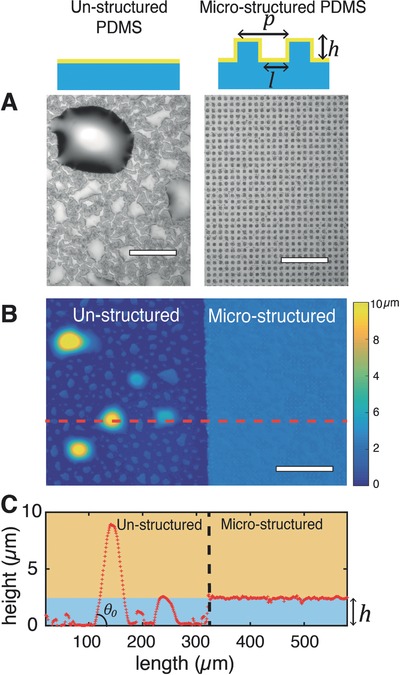
Effect of microstructured substrate on Ga thin film topology. A) Microscope images of thin films as deposited on unstructured (left) or microstructured (right, *h* = 2.5 µm, *l* = 4 µm, *p* = 8 µm) poly(dimethylsiloxane) (PDMS) substrate. Ga accumulates in large bulges on unstructured surface resulting in a highly inhomogeneous film while the liquid metal fills the microstructures by imbibition to form a smooth film with controlled thickness. Scale bars: 60 µm. B) Interferometric optical profilometer image of the surface of the Ga film deposited on mix unstructured and microstructured (*h* = 2.5 µm, *l* = 4 µm, *p* = 8 µm) PDMS substrate. Scale bar: 150 µm. C) Cross‐section profile along the dotted line indicated on the interferometer image displayed in (B).

In this study, we combine substrate microstructuring with lyophilic precoating to engineer Ga superlyophilic substrates. Such surfaces yield smooth and continuous Ga thin films of tailored thickness and electrical resistance. We compare wetting conditions based on pattern selection of the microtexture, and establish a model of the film sheet resistance as a function of the texture geometrical parameters.

Poly(dimethylsiloxane) (PDMS) substrates are decorated with an array of square micropillars prepared by soft lithography^17^ then coated with a 60 nm of Au film by sputtering. Next Ga is thermally evaporated. The substrate microstructure can be optimized so that its imbibition by the condensing Ga becomes thermodynamically more favorable than the formation of liquid drops.^18^ Figure 1 displays a flat Ga film evaporated on such substrate. The thickness of the film is uniform and controlled by the height of the micropillars (Figure 1C).

We assess the topology and the root mean square (RMS) roughness (Rq) of the Ga film deposited on flat and microstructured PDMS substrates using an interferometric profilometer (Figure 1B,C). The micropillars are characterized by their height (*h*), interpillar distance (*l*), and pitch (*p*). On unstructured substrate, Ga forms an inhomogeneous film (Rq = 1.6 µm). The majority of the liquid Ga accumulates in drops displaying an averaged contact angle of 22.9° ± 4.6° (mean ± std, *n* = 10) (Figure S1, Supporting Information). By contrast, on microstructured substrate (*h* = 2.5 µm, *l* = 4 µm, and *p* = 8 µm), Ga spreads in between the pillars, fills the open volume (to the thickness *h*) and forms a smooth film (Rq = 84 nm). The normalized projected area *A* of the liquid Ga film (i.e., the projected area excluding the pillars) is given by following equation

(1) $$A=\frac{\left(2pl-l^{2}\right)}{p^{2}}$$

Increasing the interpillar distance *l* and pitch *p* maximizes the film's projected area but eventually leads to dewetting conditions (Figure S2, Supporting Information). Moreover, in the absence of Au layer, thermal evaporation of Ga on bare microtextured PDMS substrate results in a nonconducting arrangement of liquid Ga microdroplets (Figure S3, Supporting Information) as previously described.^15^

Next, we explored a range of geometrical features of the microtexture leading to a smooth (flat) film. Bico et al.^18^ established the conditions for imbibition of a liquid drop into a microstructured substrate. Similarly to their results, we observed that depending on the aspect ratio of the pillar (*h*/*l*) and the wetting properties on a unstructured substrate (θ_0_), the condensation of evaporated Ga on the superlyophilic substrate can either lead to a smooth film by imbibition of the pattern when $\tan(\theta_{0})<\frac{h}{l}$ or dewetting and formation of drops when $\tan(\theta_{0})>\frac{h}{l}$ (**Figure** **2**). An aspect ratio *h*/*l* close to 0.42 maximizes the projected surface of Ga.

**Figure 2:**
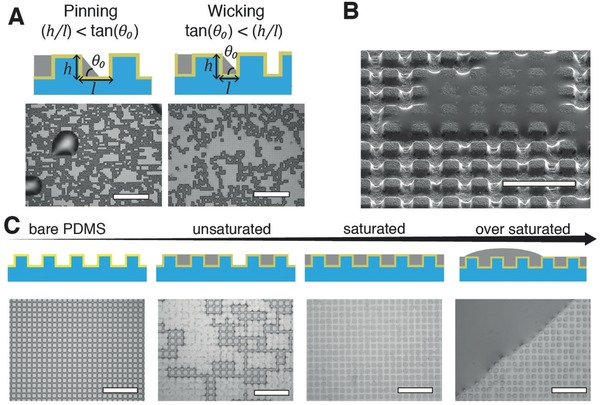
Growth of biphasic Au/Ga thin films on microstructured PDMS membranes. A) Influence of the microstructure geometrical parameters on imbibition regime. Optical microscope images of Ga film in a “pinned” metastable state (left, *h* = 1.5 µm, *l* = 4 µm, *p* = 8 µm) and in a “wicking” state (right, *h* = 2.5 µm, *l* = 4 µm, *p* = 8 µm). Scale bars are 150 µm. B) SEM image of an unsaturated of microstructured PDMS substrate (*h* = 2.5 µm, *l* = 4 µm, *p* = 8 µm). Scale bar is 20 µm. C) Schematic representations and optical microscope images of the growth of the Ga film on microstructured PDMS substrate (*h* = 1.5 µm, *l* = 2 µm, *p* = 6 µm). Scale bar: 30 µm.

We further characterized the growth of the Ga film when the imbibition criterion was verified (Figure 2C). Only a finite amount of liquid Ga can accumulate within the grooves of the microstructured substrate. We considered the pattern to be saturated when Ga occupied the whole volume in between the pillars. Under the saturation point, the dispersion of Ga was not homogenous. By capillarity, the liquid Ga rearranged to form a stochastic dispersion of coalesced Ga clusters (Figure 2B). Increasing the amount of Ga up to the point of saturation resulted in a smooth film. Above the saturation point, the excess liquid metal aggregated in large macroscopic drops (>500 µm in diameter) sitting on top of the saturated substrate.

We next evaluated the electromechanical performance of the obtained saturated films during uniaxial stretching. Test tracks (15 mm × 0.5 mm) were uniaxially stretched (0–0.8 strain) while their electrical resistance was recorded using a four‐point probe set up. Flat Ga films (on microstructured substrate) displayed significantly lower resistance compared to the films on unstructured control; both film types reversibly stretched to large strain (**Figure** **3**A). Figure 3B shows a scanning electron microscopy (SEM) image of a Ga film on microstructured substrate under 50% of applied engineered strain. The liquid Ga follows the uniaxial deformation spreading in‐between the pillars but remaining as a continuous and homogenous film.

**Figure 3:**
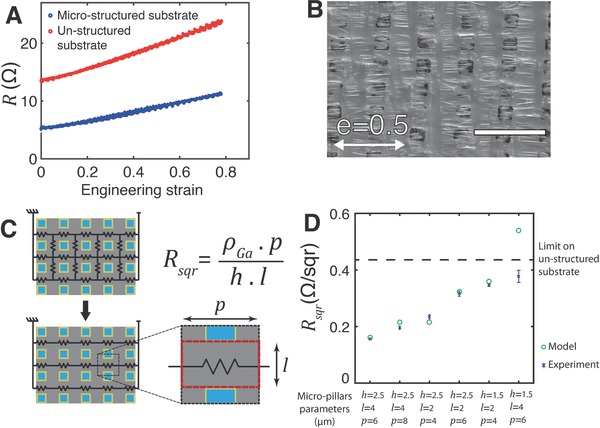
Electromechanical response of Ga films on microstructured superlyophilic substrates under large uniaxial deformation. A) Change of electrical resistance as a function of applied engineering strain *e* of biphasic conductive tracks (15 × 0.5 mm^2^ length and width) on microstructured (blue, *h* = 2.5 µm, *l* = 6 µm, *p* = 8 µm)) and flat (red) PDMS substrate. B) SEM images of Ga film on microstructured substrate (*h* = 2.5 µm, *l* = 4 µm, *p* = 8 µm) under 50% applied engineered strain. Scale bar is 20 µmC). Equivalent electrical circuit and theoretical sheet resistance as function of geometrical parameters of the micropillars. D) Theoretical end experimentally measured sheet resistance of Ga film on different microstructured substrates (*n* = 3 per condition).

We modeled the electrical resistance of the saturated film by a network of resistors as depicted Figure 3C. Due to the symmetry of the system, every node sharing the same abscissa along the principal axis has the same potential. As a result, the resistors perpendicular to the main axes can be neglected. The theoretical sheet resistance of the film at the relaxed state then corresponds to the resistance of a unit resistor from the grid and is given by the following equation

(2) $$R_{\mathrm{sqr}}=\frac{\rho_{\mathrm{Ga}}p}{hl}$$

where ρ_Ga_ and *p*, *h*, and *l* are the electrical conductivity of Ga and the geometrical parameters of the pillars array, respectively. Consequently, the sheet resistance of the film can be tailored to a specific value by choosing the right geometrical parameters. We confronted our model to our experimental measurements of the sheet resistance for six different micropillars design (Figure 3D). The model showed a good match with the experimental measurements except for one sample approaching the resolution limit of the used lithography process.

In addition, we observed the Ga films prepared on a superlyophilic substrate could withstand significantly higher electrical currents (**Figure** **4**) than their counterparts prepared on flat substrate. DC currents in 20 mA steps were applied through Ga conductors (15 × 0.5 mm^2^, *n* = 3) prepared on structured (blue) and unstructured (red) substrates. The top surface temperature was monitored with at thermal camera. At 160 mA applied current, the conductor on unstructured substrate failed (*R* → ∞) while the surface temperature rose up to Δ*T* = 150 °C. Inspection of the tracks with an optical microscope (Figure 4B) showed depletion of liquid Ga attributed to electromigration^19^ at the anodic edge of the Ga drops. Indeed, current concentration at the edges of the drops can be more than twice that across the rest of the conductor (Figure S4, Supporting Information).

**Figure 4:**
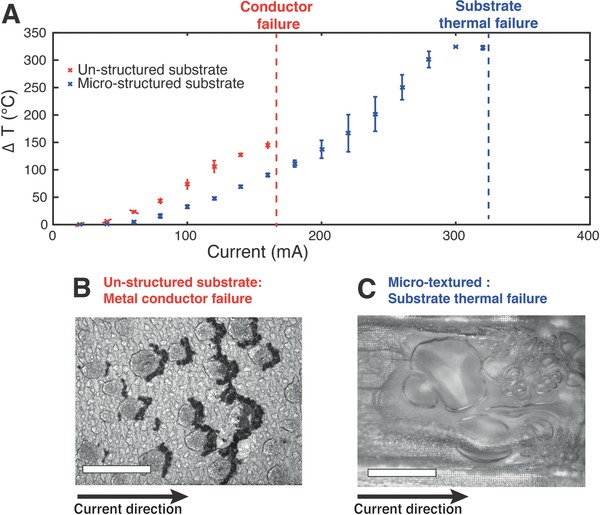
Effect of microstructured substrate on self‐heating (Joule effect) of Ga thin films. A) Temperature difference to room temperature as a function of electrical current flowing through a self‐heated Ga thin film on microstructured (blue, *h* = 2.5 µm, *l* = 4 µm, *p* = 8 µm) or unstructured (red) PDMS substrate. Test tracks are 15 mm long and 0.5 mm wide. B) Optical microscope images taken at the electrical failure point (160 mA current) of a Ga thin film on unstructured PDMS substrate. The damages in the metal conductor are located at the transition between Ga bulges and thinner part of the film. Scale bar: 200 µm. C) Optical microscope images of the failure point of a Ga thin film on microstructured PDMS substrate after running 320 mA. PDMS substrate failed due to overheating. Scale bar: 200 µm.

By contrast, the film deposited on the superlyophilic sustained a maximal current of up to 320 mA that caused the PDMS substrate to burn due to the extreme rise in temperature (Δ*T* > 300 °C), and eventually triggered failure of the conductor (Figure 4C).

In summary, we introduced a deposition and patterning technique taking advantage of wetting phenomenon to form smooth Ga thin films of controlled thickness on elastomeric substrates. Thermal evaporation of Ga prevents oxide skin formation during deposition while microstructuration of the substrate overcomes the high surface tension of Ga to form extended films below the capillary length. Compared with peer approaches, our process enables to form smooth film with tailored thickness and electrical properties while maintaining excellent electromechanical performance. The resulting liquid metal thin films open the route for high‐resolution patterning of liquid metals and more repeatable and controlled stretchable electrical conductors.

## Experimental Section

*Microstructured Mould Preparation*: 4 in. silicon wafers were exposed to Bis(trimethylsilyl)amine and spin‐coated to form 2–5 µm thick photoresist film (AZ‐1512 from MicroChemicals) and then cured at 100 °C for 60 s. Next, the resist was exposed with a 180 mJ cm^−2^ UV dose (MLA maskless aligner Heideleberg), developed in diluted AZ‐400‐K and dried. The wafer was then exposed to a short plasma and treated with a self‐assembled layer of trichloro(1H,1H,2H,2H‐perfluorooctyl) silane (Sygma‐Aldrich) in a dessicator.

*Gallium Superlyophilic PDMS Substrate Preparation*: PDMS (Sylgard 184, Dow Corning, mixed at 10:1 w:w, prepolymer:crosslinker) was spin‐coated on a microstructured mould (500 RPM for 1 min) and cured at 80 °C for at least 2 h in a convection oven. The PDMS layer was then pilled‐off from the mould and manually transferred, with structured surface facing up, to a 4 in. carrier wafer. A customized Kapton shadow mask patterned with desired layout was aligned and laminated on the microstructured PDMS substrate. Then, 60 nm of Au was sputtered through the shadow mask (AC 450, Alliance Concept).

*Gallium Evaporation*: A mass of pure Ga ranging from 0.5 to 2 g was thermally evaporated (E300, Alliance Concept, 10^−6^ mbar) on the gallium superlyophilic substrate. After evaporation, the Kapton mask was delicately peeled off the substrate to form the desired pattern.

*Interferometric Profilometer*: Interferometric profilometer data were acquired using a ContourGt profilometer from Bruker an OLS 4100 from Olympus and further analyzed with Vision 64 software and custom matlab code.

SEM: SEM images were acquired in an SU5000 microscope from Hitachi, using the secondary electron detector at a beam energy of 5 keV.

*Test Samples Preparation*: Ga conductors were stencil‐patterned on 120 µm superlyophilic PDMS substrate to produce 15 mm long and 0.5 mm wide electrical conductors. Samples were subsequently cut in 10 mm by 30 mm rectangles and peeled from the wafer.

*Uniaxial Electromechanical Characterization*: Test samples were mounted on a custom‐built uniaxial tensile stretcher programmed to actuate two clamps moving in opposite directions along the horizontal plane. Each clamp featured two contact pads that provided constant electrical and mechanical contact to the sample under test. The position of the clamps and the electrical resistance of the conductor, measured using a four‐point probe method (2400 sourcemeter, Keithley), were acquired synchronously at 3.8 Hz on a computer running a dedicated LabVIEW program.

*Thermal Test*: Test samples were clamped in the uniaxial stretcher. Electrical resistance and current running through of the conductor were measured using a four‐point probes method (2400 sourcemeter, Keithley) and acquired synchronously at 3.8 Hz on a computer running a custom LabVIEW program. In parallel, a thermal camera (A325 sc, FLIR systems) and close up lens (1 × 25 µm, FLIR systems) were used to monitor synchronously the temperature at the surface of the samples (sampling rate: 60 Hz).

## Conflict of Interest

The authors declare no conflict of interest.

## Supporting information

Supplementary
