# Supplementary material for: A Method to Form Smooth Films of Liquid Metal Supported by Elastomeric Substrate
Source: Adv Sci (Weinh). 2018 Aug 9;5(10):1800256. doi: 10.1002/advs.201800256 (PMC6193177; doi:10.1002/advs.201800256)
Supplement: Supplementary file 1 — Supplementary [file ADVS-5-1800256-s001.pdf]

Copyright WILEY-VCH Verlag GmbH & Co. KGaA, 69469 Weinheim, Germany, 2018.

ADVANCED SCIENCE
Open Access

Supporting Information

for *Adv. Sci.,* DOI: 10.1002/advs.201800256

A Method to Form Smooth Films of Liquid Metal Supported by Elastomeric Substrate

*Arthur Hirsch and Stéphanie P. Lacour**

Copyright WILEY-VCH Verlag GmbH & Co. KGaA, 69469 Weinheim, Germany, 2016.

Supporting Information

Engineered Gallium super-lyophilic substrates: application to stretchable conductors

*By Arthur Hirsch and Stéphanie P. Lacour**

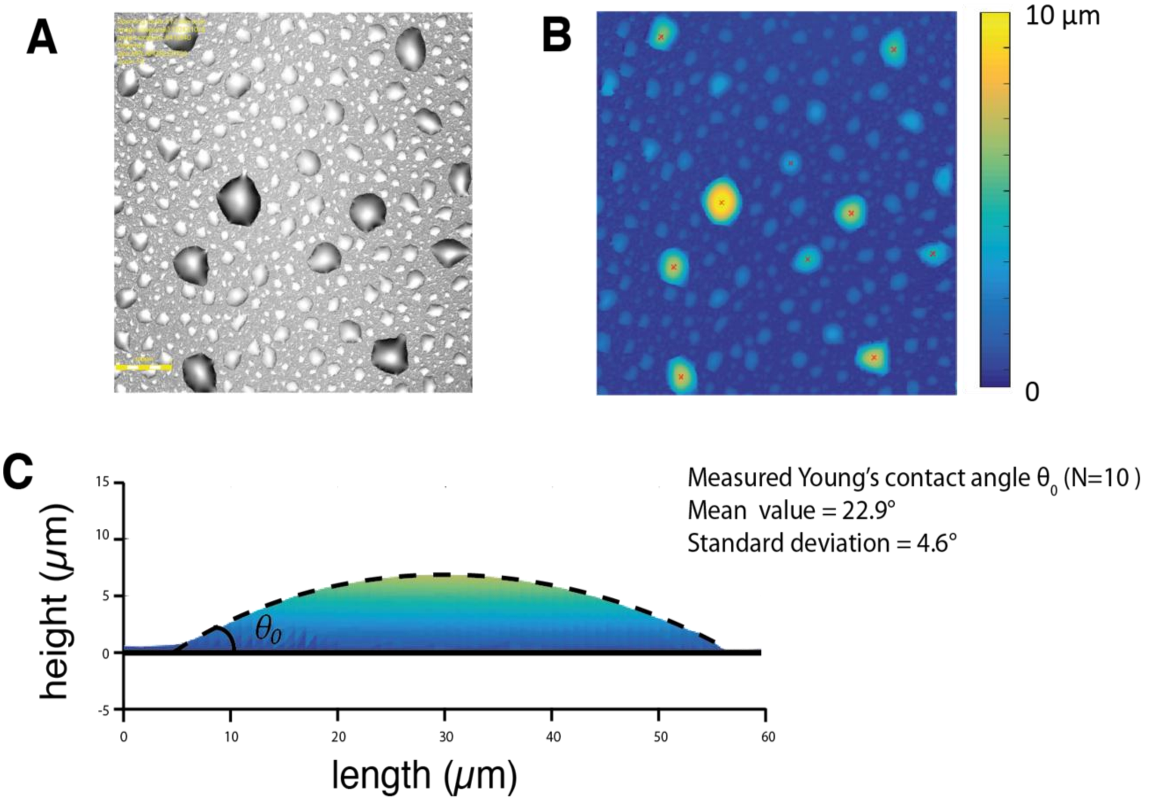

Figure S1. Wetting angle of gallium drops on un-structured lyophilic PDMS. A) and B) Optical image and laser profilometer image of the surface of the gallium film deposited on un-structured lyophilic PDMS. Drops identified by the red marks were used to compute Young's

contact angle θ_0. Scale bars: 100 µm. C) Cross section profile of a drop of gallium on un-structured lyophilic PDMS. The dashed line represents the circular interpolation of the drop. The angle at the intersection of the circular intersection and the surface was then measured to compute the young contact angle: 22.9°±4.6° (mean ± std, n=10).

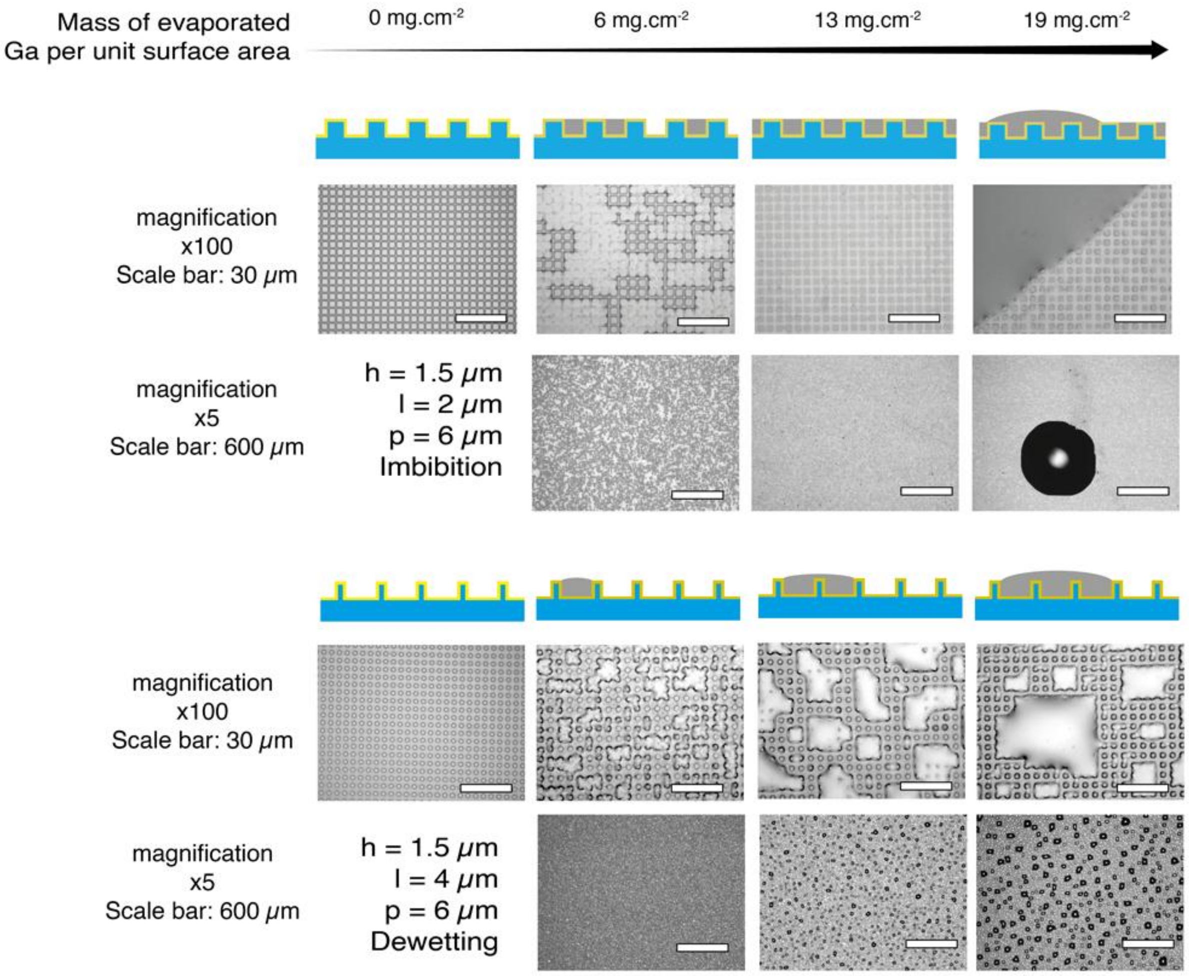

Figure S2: Influence of the micro-structure geometrical parameters on imbibition regime. Growth of Ga film on two different micro-structured PDMS substrates (top: dense array, l = 4µm and bottom: more “open” array, l = 2µm) illustrating imbibition (top) and dewetting (bottom) regimes.

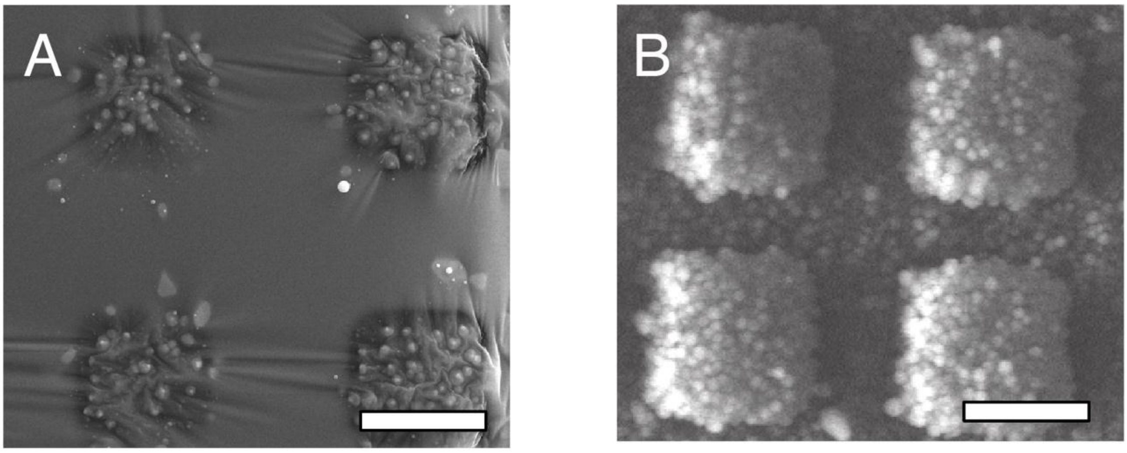

Figure S3: Influence of the gold coating. SEM images of the Ga film evaporated on micro-structured PDMS substrate (h = 1.5 µm, l = 4 µm, p = 8 µm). A) The substrate is coated with a 60 nm thick sputtered Au film prior to Ga evaporation. The thin Au coating allows for the Ga to spread on the surface by diffusion and alloying, and to fill the open volume in between the pillars; the top of the pillars is covered by a thin $AuGa_2$ intermetallic film. Scale bar: 4 µm B) On bare micro-structured PDMS, the evaporated gallium condenses on the substrate to form a non-percolating layer of micro-droplets. Scale bar: 4 µm.

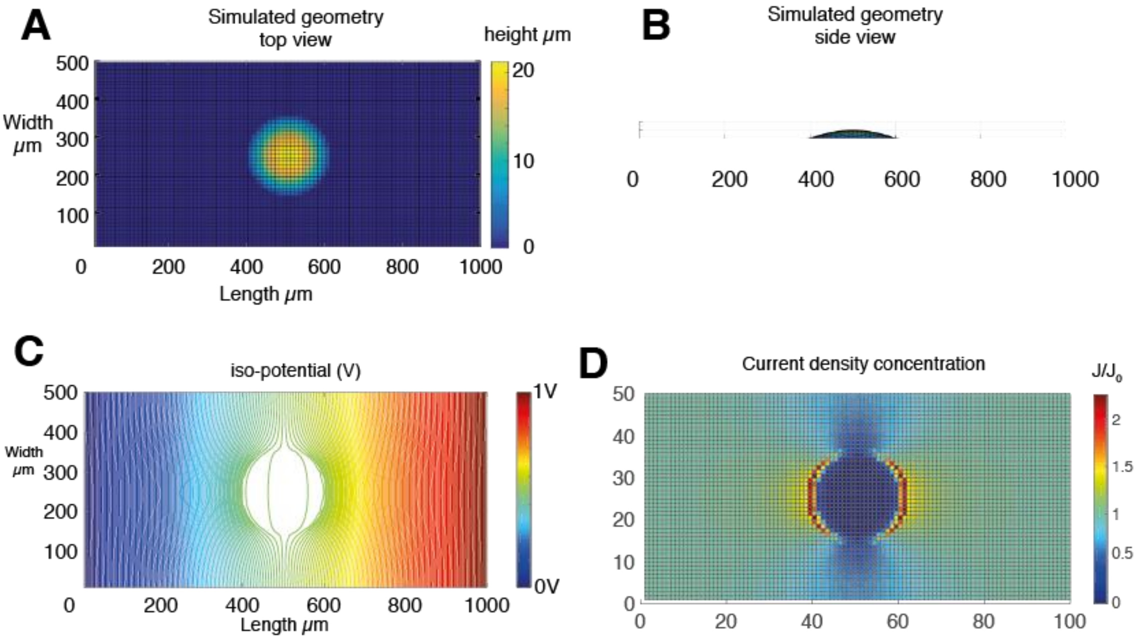

Figure S4: Numerical simulation of the current concentration at the edges of the gallium drops in a gallium film on un-structured lyophilic substrate. A) & B) Top and side view of the simulated geometry. The geometry consists of a 500 µm wide and 1000 µm long film with a thickness of 300 nm. The film has a 21 µm drop shape protrusion in his center forming spherical cap (23° contact angle). The geometry is meshed by regular hexahedron elements with a 10 µm square base and a height of 0.3 µm. Dirichlet-type boundary conditions to the potential are applied to the left and right extremities of the film and solve the potentials using the same method detailed previously [20]. C) Iso-potential distribution in the film D) Normalised current density. Current density at the vicinity of the drop can be twice more important than in the rest of the film.
